# Fetal cardiac cine imaging using highly accelerated dynamic MRI with retrospective motion correction and outlier rejection

**DOI:** 10.1002/mrm.26686

**Published:** 2017-04-03

**Authors:** Joshua F.P. van Amerom, David F.A. Lloyd, Anthony N. Price, Maria Kuklisova Murgasova, Paul Aljabar, Shaihan J. Malik, Maelene Lohezic, Mary A. Rutherford, Kuberan Pushparajah, Reza Razavi, Joseph V. Hajnal

**Affiliations:** ^1^ Division of Imaging Sciences and Biomedical Engineering King's College London London United Kingdom; ^2^ Department of Congenital Heart Disease Evelina Children's Hospital London United Kingdom; ^3^ Centre for the Developing Brain King's College London London United Kingdom

**Keywords:** magnetic resonance imaging, fetal heart, cardiac cine, motion correction, congenital heart disease

## Abstract

**Purpose:**

Development of a MRI acquisition and reconstruction strategy to depict fetal cardiac anatomy in the presence of maternal and fetal motion.

**Methods:**

The proposed strategy involves i) acquisition and reconstruction of highly accelerated dynamic MRI, followed by image‐based ii) cardiac synchronization, iii) motion correction, iv) outlier rejection, and finally v) cardiac cine reconstruction. Postprocessing entirely was automated, aside from a user‐defined region of interest delineating the fetal heart. The method was evaluated in 30 mid‐ to late gestational age singleton pregnancies scanned without maternal breath‐hold.

**Results:**

The combination of complementary acquisition/reconstruction and correction/rejection steps in the pipeline served to improve the quality of the reconstructed 2D cine images, resulting in increased visibility of small, dynamic anatomical features. Artifact‐free cine images successfully were produced in 36 of 39 acquired data sets; prolonged general fetal movements precluded processing of the remaining three data sets.

**Conclusions:**

The proposed method shows promise as a motion‐tolerant framework to enable further detail in MRI studies of the fetal heart and great vessels. Processing data in image‐space allowed for spatial and temporal operations to be applied to the fetal heart in isolation, separate from extraneous changes elsewhere in the field of view. Magn Reson Med 79:327–338, 2018. © 2017 The Authors Magnetic Resonance in Medicine published by Wiley Periodicals, Inc. on behalf of International Society for Magnetic Resonance in Medicine. This is an open access article under the terms of the Creative Commons Attribution License, which permits use, distribution and reproduction in any medium, provided the original work is properly cited.

## INTRODUCTION

Magnetic resonance imaging increasingly is being used as an adjunct to ultrasound to assess the developing fetus. Motion, however, remains a key limiting factor to the use of MRI to depict the fetal heart and great vessels in utero 1, 2, 3. The challenges are numerous when imaging a small, rapidly beating heart that is subject to various regular and spontaneous movements within the context of the maternal torso.

The fetal heart has a complex structure and is a relatively small and dynamic target for MRI. The ventricles are each only 15 mm in diameter in late gestation 4, and the normal fetal heart rate is 120 to 160 beats per minute 5, with low variation compared to adults 6 and no significant change in heart rate pattern during MRI 7. Gas exchange to the fetus occurs through the placenta, but episodic fetal respiratory movements still occur, causing displacement of the fetal diaphragm and chest wall 8. The fetus also can move freely, and general fetal movements occur at irregular intervals. Overall mobility is reduced with increased occupancy of the uterus at later gestational ages, although movement of the fetal trunk is present throughout gestation 9, 10. Maternal respiration also is a factor because movement of the maternal anatomy leads to potentially large displacements of the entire fetal body. Although these sources of motion can be minimized during scanning, for example by maternal breath‐hold or fetal sedation, such approaches can be both impractical and unacceptable and are likely to cause maternal discomfort and anxiety.

Synchronization with the fetal cardiac cycle during MRI data acquisition also poses a challenge. Electrocardiogram (ECG) gating of the fetal heart is unreliable 11, but segmented cine acquisitions have been achieved using self‐consistency in reconstruction to infer a gating signal 12, 13 or MR‐compatible Doppler ultrasound‐based triggering 14, 15. However, with these methods, both regular and spontaneous motion may still corrupt the data.

In this work, we aim to develop a MRI acquisition and reconstruction strategy to depict fetal cardiac anatomy in the presence of maternal and fetal motion. Retrospective reconstruction of cine images from a series of dynamic (real‐time) images acquired without breath‐hold or ECG triggering has been investigated in adult cardiac MRI 16. In the context of fetal cardiac imaging, a dynamic acquisition may provide serial views of the fetal heart and surrounding anatomy fast enough to freeze the various types of expected motion with the potential for retrospective processing to detect movement and correct for its effects. However, for in utero examinations, real‐time imaging cannot achieve the same resolution as a segmented cine reconstruction (when cardiac gating is feasible), resulting in compromised signal‐to‐noise ratio (SNR) and resolution. To overcome these limitations, we explored an imaging strategy that favors both spatial and temporal fidelity at acquisition and initial reconstruction, resulting in noisy real‐time images that then can be compounded to recover SNR while generating a cine image series representing a single cardiac cycle 17. In this work, we have developed the idea further, producing a pipeline approach that starts with highly accelerated dynamic MRI of the fetal heart and uses retrospective, image‐based techniques to provide cardiac synchronization, motion correction, and outlier rejection to generate a fully corrected 2D cine image series. The method has been evaluated on 30 fetuses.

## METHODS

**Figure 1:**
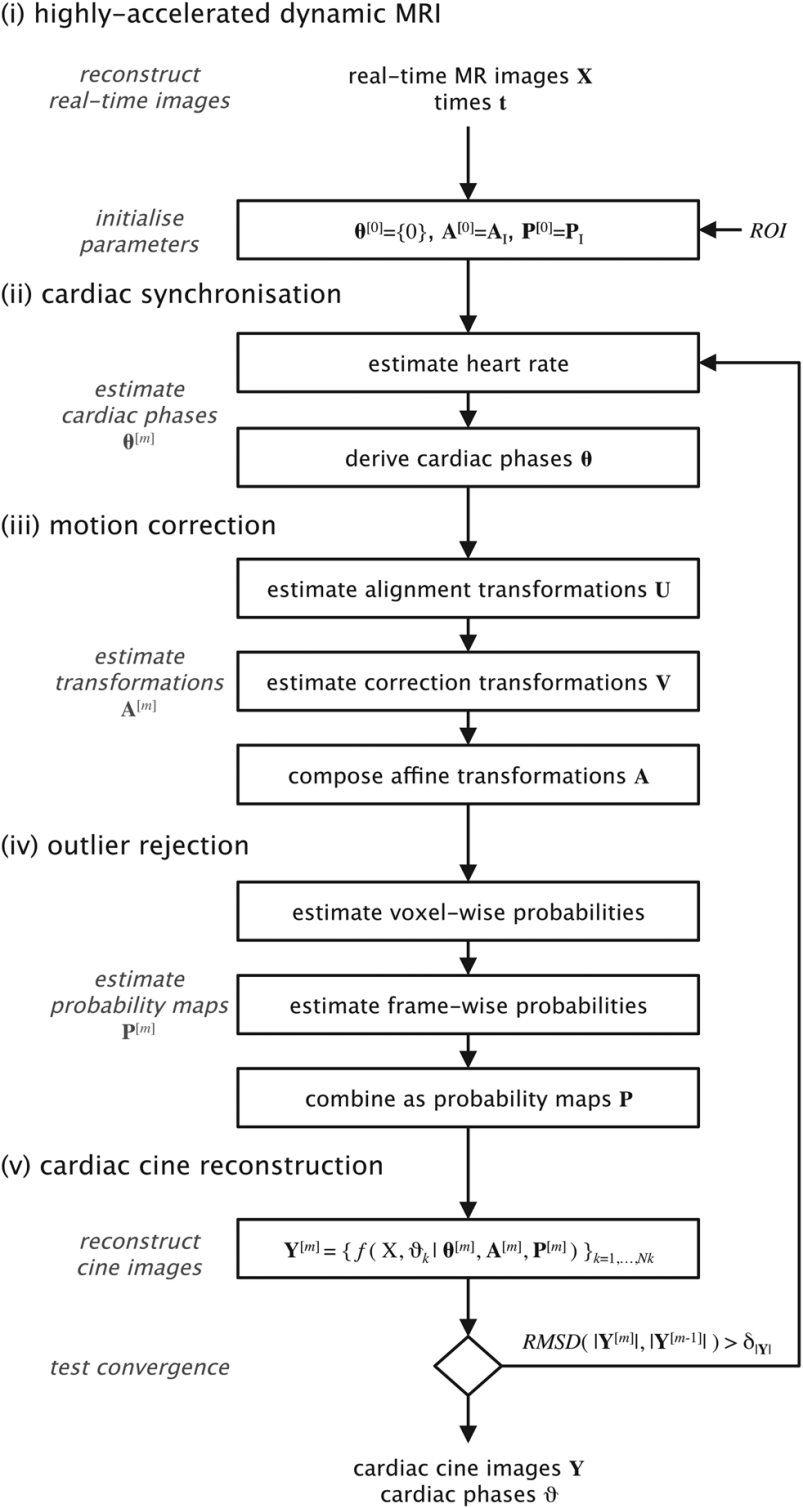
Proposed method for motion‐tolerant fetal cardiac cine imaging. The steps comprise (**i**) data acquisition and image reconstruction of time series of highly accelerated dynamic (real‐time) MR images, X, followed by retrospective, image‐based (**ii**) cardiac synchronization, (**iii**) motion correction, and (**iv**) outlier rejection, before a final (**v**) cardiac cine reconstruction. Steps (ii)–(v) were repeated using updated parameters, θ, A, and P, until the RMSD of the signal intensity in the region of interest of successive cardiac cine reconstructions, Y, was below the tolerance $\delta_{\left|Y\right|}$. ROI, region of interest; RMSD, root mean square difference.

The proposed strategy involves 1) acquisition and reconstruction of highly accelerated dynamic MRI, followed by 2) cardiac synchronization, 3) motion correction, 4) outlier rejection, and finally 5) cardiac cine reconstruction. Processing of this data was performed sequentially and iteratively, as depicted in Figure 1. The entire reconstruction pipeline was implemented in MatLab R2016a (Mathworks, Natick, USA) using methods of the signal processing, image processing, and statistics toolboxes. A repository of source code is available: https://github.com/jfpva/fetal_cmr_cine_2d (SHA‐1:556b9646bf).

Thirty singleton pregnancies (25–35 weeks gestational age) were scanned on a 1.5 Tesla Ingenia system (Philips, Best, Netherlands), including seven volunteers and 23 cases with congenital heart disease and related conditions. Scans were performed in one or more views of the fetal heart, typically short‐ and long‐axis orientations without maternal breath‐hold. Expectant mothers were scanned in a left lateral tilt position using an anterior torso coil array in combination with a posterior spine coil array to measure signal in 28 receiver channels. Studies were conducted with the approval of the local research ethics committee, and all participants gave written informed consent prior to enrollment. Reconstruction methods were established using data from five cases collected early in the study. Reconstruction parameters were refined further, once all cases were scanned using an additional five data sets selected at random.

The following sections outline the conceptual framework of this approach, beginning with a description of the acquisition and reconstruction of highly accelerated dynamic MRI and subsequent kernel‐weighted interpolation to reconstruct cine images, followed by the details of cardiac synchronization, motion correction, and outlier rejection.

### Highly Accelerated Dynamic MRI

**Figure 2:**
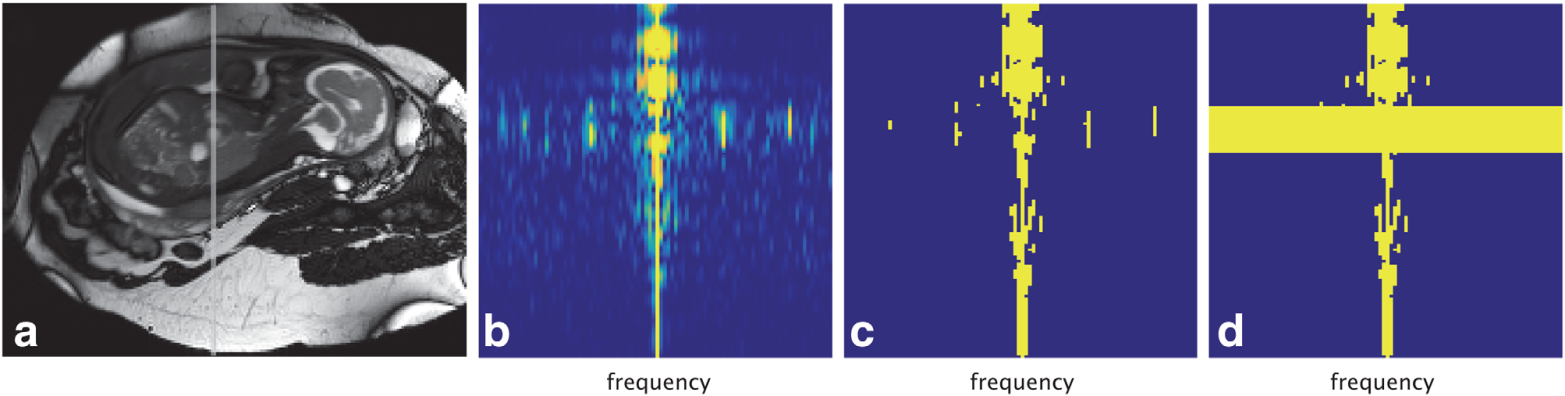
A 33‐week gestational age fetus in utero surrounded by maternal anatomy in (**a**) the signal intensity of the time‐averaged baseline estimate, $\bar{\rho }$, of the *k‐t* sampled dynamic data. (**b**) The *x‐f* prior, Θ, at the location of the gray vertical line in (a) reveals spatiotemporal sparsity in the x‐f domain. Regions of the *x‐f* prior above the regularization term (ie, $\Theta >\Lambda S\Psi S^{H}{\left(SS^{H}\right)}^{-2}$) are shown for (**c**) spatially uniform regularization with $\Lambda =\lambda_{0}\;I$, and (**d**) spatially adaptive regularization, in which Λ has diagonal elements $\lambda_{j\notin ROI}=\lambda_{0}$ and $\lambda_{j\in ROI}=\lambda_{ROI}$, with $\lambda_{0}>\lambda_{ROI}$.

The fetal body in utero is surrounded by the maternal uterus and torso. The discrepancy in size and temporal dynamics of the fetal heart and surrounding anatomy leads to highly complementary signal properties in space and time, with the fetal heart occupying only a small fraction of the spatial field of view but exhibiting a large range of temporal frequencies, *f*, whereas the surrounding anatomy is much more slowly varying. Consequently, when a dynamic sequence designed with time resolution suitable to capture the pulsation of the fetal heart is Fourier‐transformed in time to create a *x‐f* representation, the resulting space has sparse signal content, as shown in Figure 2b. Real‐time imaging with *k‐t* undersampling can take advantage of this spatiotemporal sparsity to achieve high acceleration factors in which k‐space is undersampled with a sheared grid pattern, resulting in aliasing of the *x‐f* support with minimal overlap between aliases.

The *k‐t* sensitivity encoding (SENSE) reconstruction aims to recover a vector of complex‐valued *x‐f* signals, ρ, for voxels aliased to $\rho_{alias}$ using prior knowledge of the spatiotemporal distribution of the unaliased signals, Θ, and receiver array sensitivities, S 18. The closed‐form *k‐t* SENSE reconstruction problem in *x‐f* space is given by

(1) $$\rho =\bar{\rho }+{\Theta S}^{H}{\left({S\Theta S}^{H}+\lambda \Psi \right)}^{-1}(\rho_{alias}-S\bar{\rho }),$$

where $\bar{\rho }$ is a time‐averaged baseline estimate of ρ, and λΨ is a regularization term comprised of controlling parameter λ and noise covariance matrix Ψ. Baseline subtraction in Equation [1] aids the reconstruction by removing the contribution of voxels dominated by static signal, reducing the number of voxels contributing aliased signal.

The priors in Θ can be determined from full field of view, low spatial‐resolution training data that is acquired interleaved with the undersampled scan or during a separate acquisition phase 18. The latter approach was adopted for this study because maximizing temporal resolution in the final images was key. The choice of λ balances content revealed by Θ, with noise properties specified in Ψ such that reducing λ recovers more dynamic content but increases the noise in the final reconstruction. In the fetal cardiac case, most of the spatial field of view contains regions of static or slowly moving anatomy, with an easily identified and highly localized region of interest ( ROI) for which there are higher temporal frequencies to recover. This situation was exploited by adopting spatially adaptive regularization in which λ was preferentially reduced in regions of highly dynamic anatomy, leading to

(2) $$\rho =\bar{\rho }+\Lambda^{-1}{\Theta S}^{H}{\left({S\Lambda }^{-1}{\Theta S}^{H}+\Psi \right)}^{-1}(\rho_{alias}-S\bar{\rho }),$$

where Λ is a diagonal matrix of spatially adaptive regularization‐controlling parameters. In this work, the elements of Λ were assigned a base value, $\lambda_{0}$, for voxels outside the ROI and a much lower value, $\lambda_{ROI}$, for voxels inside the ROI, as shown in Figures 2c through d. Using data collected in preparation for this study, a regularization level of $\lambda_{0}$ = 0.0014 was found to capture the dynamics of the maternal anatomy and limit noise in the reconstructed real‐time images. A highly permissive regularization level of $\lambda_{ROI}=0.01\;\lambda_{0}$ was used to preserve the full temporal resolution of the accelerated acquisition, as indicated in Figure 2d. In this application, reduction of λ was well tolerated because noise in the real‐time images was reduced when those images were combined to generate the final cine images.

MRI is generally considered safe at 1.5 T in the second and third trimester 19, but caution should be exercised to limit RF exposure 20 and acoustic noise levels 21. Imaging was performed with a 2D Cartesian balanced steady‐state free precession (bSSFP) sequence, as it is the standard for postnatal cardiac cine MR, and the combination of short acquisition and high signal has been shown to be effective for imaging the fetal heart 22. Uniform‐density *k‐t* sampling with an optimal spatiotemporal grid pattern 23 was used to minimize the overlap of aliases in *x‐f* space. Coil calibration data was acquired in a prescan, and low spatial‐resolution training data was acquired immediately following the undersampled data. Minimal overlap between aliases of the *x‐f* support was maintained by setting the time taken to phase‐encode a field of view just large enough to encompass the fetal heart, $t_{enc}$, as the maximum frame rate. When viewed from the perspective of acceleration by undersampling, the upper limit on the acceleration factor was set by the ratio of the full maternal field of view in the phase‐encode direction to the size of the fetal heart. Imaging parameters were selected with reference to previous MRI studies of the fetal heart using segmented cine 12 and real‐time 24 bSSFP, and were optimized to yield adequate signal and contrast to depict the fetal heart and surrounding anatomy with sufficient temporal resolution to capture cardiac pulsation during preliminary pilot cases not included in this report 25. Single‐slice imaging was performed with the following sequence parameters: repetition time (TR)/echo time (TE) 3.8/1.9 ms; flip angle 60 °; field of view 400 × 304 mm; voxels 2 × 2 × 6 mm; acceleration factor 8; and temporal resolution 72 ms. Operation was constrained to 2 W/kg or less whole body‐specific absorption rate, and low peripheral nerve stimulation and gradient‐induced acoustic noise settings were used. This limited scanner performance resulting in variations in timing, with TR ranging from 3.8 to 4.4 ms (median 4.2 ms) and temporal resolutions ranging from 72 to 83 ms (median 81 ms). In five cases, the field of view was increased to accommodate maternal anatomy, leading to temporal resolutions 1 or 2 TR longer.

In initial cases, a scan duration of 16 to 20 seconds was used to establish the amount of data typically required for robust real‐time and cine reconstructions. This data was reconstructed at full and reduced durations, showing increased image quality with increased scan duration but at the same time a higher chance of motion corruption. A scan duration of approximately eight seconds was determined to balance the benefits and risks. All fetal real‐time images were reconstructed with an equivalent scan duration of approximately 8 seconds.

An initial *k‐t* SENSE reconstruction with spatially uniform regularization ( $\lambda =\lambda_{0}$ in Equation 1) was used as a reference to specify the static ROI delineating the margin of the fetal heart. This was the only manual intervention required for the complete postprocessing pipeline. Real‐time images were then reconstructed using Equation [2], following the process described in the original *k‐t* SENSE method 18.

### Cine Reconstruction from Dynamic MRI

Acquisition and reconstruction of highly accelerated dynamic MRI results in a time series of real‐time images $X={\left\{X_{i}\right\}}_{i=1,\ldots ,N_{i}}$ at times $t={\left\{t_{i}\right\}}_{i=1,\ldots ,N_{i}}$, where i indexes real‐time frame number from 1 to $N_{i}$ = 96, and frame $X_{i}$ has elements $x_{j,i}$ at 2D spatial coordinates j.

If the heart rate is known, retrospective cardiac synchronization can be performed by mapping times t to cardiac phases $\theta ={\left\{\theta_{i}\right\}}_{i=1,\ldots ,N_{i}}$, where the values of $\theta_{i}$ fall on the cyclic interval [0,2π]. Reordering the real‐time image series according to $\theta_{i}$ leads to a densely sampled set of images for a single cardiac cycle. Real‐time images were kept as complex valued data arrays to facilitate further processing.

Kernel‐weighted interpolation can be used to reconstruct a cine image, $Y_{\vartheta }$, at cardiac phase ϑ from a combination of $X_{i}$ as:

(3) $$Y_{\vartheta }=\frac{\sum_{i}d_{i}X_{i}}{\sum_{i}d_{i}},$$

where weights $d_{i}$ are obtained from a kernel function $d(\vartheta ,\theta_{i})$ that acts as a temporal point spread function, distributing the values of X to $Y_{\vartheta }$. In the *k‐t* SENSE method, X is obtained by inverse Fourier transform of the reconstructed temporal frequency spectrum, which strictly is band‐limited by the sampled frame rate; and data for a single frame are considered acquired simultaneously, which suggests that the appropriate kernel function is a sinc. However, the cyclic nature of the cardiac phase requires that the kernel width does not exceed 2 π and that the kernel weight should be smoothly varying at ±π. In this work, a Tukey window was used to taper the kernel near ±π, leading to a kernel‐weighting function of the form

(4) $$d(\vartheta ,\theta_{i})=sinc\left(\pi \left(\frac{∠\left(\vartheta ,\theta_{i}\right)}{\theta_{enc}}\right)\right)win_{Tukey}\left(∠\left(\vartheta ,\theta_{i}\right)|\alpha \right),$$

where $∠(\vartheta ,\theta_{i})$ is the angular difference between ϑ and $\theta_{i}$ wrapped on the interval [−π,+π]; the temporal resolution of the acquisition is given by $\theta_{enc}=2\pi \;t_{enc}/t_{RR}$ in units of cardiac phase, using the cardiac period $t_{RR}$ to normalize $t_{enc};$ and α is the proportion of the outer edge of the window with tapered cosine lobes.

Motion correction and outlier rejection were included in the reconstruction of $Y_{\vartheta }$ to improve image quality in the presence of fetal and maternal motion. The aim of motion‐correction was to align the position of the fetal heart across all $X_{i}$. A set of spatial transformations, $A={\left\{A_{i}\right\}}_{i=1,\ldots ,N_{i}}$, was estimated using image registration techniques, with transformation of real‐time image frame $X_{i}$ by $A_{i}$ denoted as $X_{i}^{\left(A\right)}$. Outlier rejection was used to reduce the influence of corrupted data, such as voxels with motion artifact or frames with inconsistent anatomical views. Posterior probability maps, $P={\left\{P_{i}\right\}}_{i=1,\ldots ,N_{i}}$, were generated to indicate the probability, $p_{j,i}$, that each voxel in X was an inlier, and were used in the cine reconstruction as a robust statistic in the kernel‐weighting function.

Including motion correction and outlier rejection in Equation [3] lead to a kernel‐weighted interpolation of $X_{i}$ at cardiac phase ϑ, given estimates of cardiac phases θ, spatial transformations A, and posterior probability maps P, of the form

(5) $$\begin{array}{rr} Y_{\vartheta } & =f(X,\vartheta |\theta ,A,P)=\underset{i}{\sum }W_{i}\circ X_{i}^{\left(A\right)} \end{array},$$

with kernel‐weighting and normalization combined in weighting maps $W={\left\{W_{i}\right\}}_{i=1,\ldots ,N_{i}}$, where ∘ indicates Hadamard (element‐wise) matrix multiplication, and superscript (A) denotes spatial transformation by A. The weighting map for real‐time image frame i is then given by

(6) $$W_{i}=\left(d_{i}P_{i}^{\left(A\right)}\right)⊘\left(\underset{i^{\prime }=1}{\overset{N_{i}}{\sum^{}}}d_{i^{\prime }}P_{i^{\prime }}^{\left(A\right)}\right),$$

where ⊘ indicates element‐wise matrix division.

Cardiac synchronization, motion correction, and outlier rejection steps were performed iteratively. Parameters were initialized for iteration m=0 such that cardiac phase was zero for all real‐time image frames, $\theta^{\left[0\right]}={\left\{0\right\}}_{i=1,\ldots ,N_{i}}$; spatial transformations were set to an identity transformation, $A^{\left[0\right]}=A_{I}$, such that $X=X^{\left(A_{I}\right)}$; and probability maps had all voxels as full inliers, $P^{\left[0\right]}=P_{I}$ with $p_{j,i}=1$ for all j and i.

### Cardiac Synchronization

Synchronization with the cardiac cycle was required to resolve the beating of the fetal heart. One advantage of the high temporal resolution acquisition was that the fetal heart rate could be estimated directly from the real‐time image series because the periodicity of the fetal heart was revealed as conspicuous peaks in the temporal frequency spectrum.

**Figure 3:**
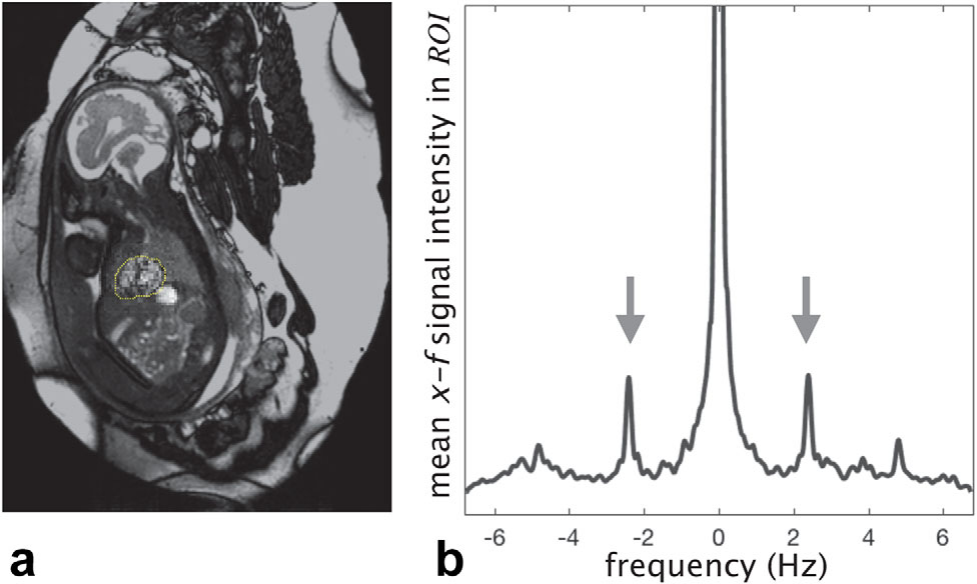
(**a**) Image frame from real‐time series reconstructed using Equation [2] corresponding to fetus shown in Figure 2a, with region of interest over the fetal heart (yellow dotted line), rotated so the fetus is in radiographic image orientation. (**b**) The fetal heart rate (arrows) appears as local maxima in the range of fundamental frequencies (1.8–2.7 Hz) of the mean *x‐f* signal intensity in the region of interest.

An estimate of the fetal cardiac period, $t_{RR}$, was obtained from the spatially transformed real‐time images, $X^{\left(A\right)}$. The temporal frequency spectrum of the fetal heart was calculated by taking the spatial mean in *x‐f* space over the ROI after interpolation to a resolution of 0.1 beats per minute by zero‐padding in time before Fourier transformation. The local maxima in this signal in the range of fundamental frequencies (1.8–2.7 Hz) was identified as the fetal heart rate corresponding to $t_{RR}$, as shown in Figure 3b. This estimate of the fetal heart rate was used to map each $t_{i}$ to an associated cardiac phase, $\theta_{i}=2\pi (t_{i}\text{mod}t_{RR})/t_{RR}$.

### Motion Correction

Rigid body in‐plane image registration was used to estimate a set of affine transformation matrices $A={\left\{A_{i}\right\}}_{i=1,\ldots ,N_{i}}$ that align the position of the fetal heart across the real‐time image series. Image registration was performed using the Matlab imregtform function (MathWorks) between pairs of source and matched target images by minimizing a sum of squares differences cost function. Confounding fetal and maternal anatomy was masked out using the ROI, which was doubled in area for image registration as inclusion of some fetal chest anatomy was found to improve results. The origin of coordinates was defined as the centroid of the ROI, and three iterations with decreasing Gaussian spatial blurring ( σ = 1.6, 1.2, 0.8 mm) of the source images were used to facilitate convergence. Spatial transformations were applied to X using cubic interpolation to yield $X^{\left(A\right)}$, whereas linear interpolation was used for $P^{\left(A\right)}$ to maintain discontinuities in the probability maps.

In some cases, an initial image registration was found to include some overfitting manifest by a periodic twisting of the cine sequence along the long axis of the heart as it beats. Thus, two image registration steps were performed for each $X_{i}$: the first registration resulted in spatial transformations U that provided most of the spatial alignment, and the second resulted in spatial transformations V that compensated for any residual twisting of the heart. These two sets of spatial transformations were then composed as $A_{i}=U_{i}V_{i}$.

Spatial transformations U were obtained from image registration of the native images $X_{i}$ to target images ${\overset{⁁}{X}}_{i}$, calculated using Equation [5] at $\theta_{i}$ given current estimates of θ, A, and P, but excluding the source image frame, $X_{i}$, so that

(7) $$\begin{array}{r} {\overset{⁁}{X}}_{i}=\overset{⁁}{f}(X,\theta_{i}|\theta ,A,P)=\underset{i^{\prime }\neq \;i}{\sum }{\overset{⁁}{W}}_{i^{\prime }}\circ X_{i^{\prime }}^{\left(A\right)} \end{array},$$

where i was also omitted from the summation in Equation [6] to calculate ${\overset{⁁}{W}}_{i^{\prime }}$.

Spatial transformations V were obtained from image registration between pairs of source and target images calculated using Equation [5] and differing only in input spatial transformation. Source images were calculated as ${\left\{\;f(X,\theta_{i}|\theta ,U,P)\;\right\}}_{i=1,\ldots ,N_{i}}$ using U as input, whereas target images were calculated as ${\left\{\;f(X,\theta_{i}|\theta ,A_{I},P)\;\right\}}_{i=1,\ldots ,N_{i}}$ using the null transformation $A_{I}$ to capture the average position of the fetal heart during the whole acquisition.

### Outlier Rejection

**Figure 4:**
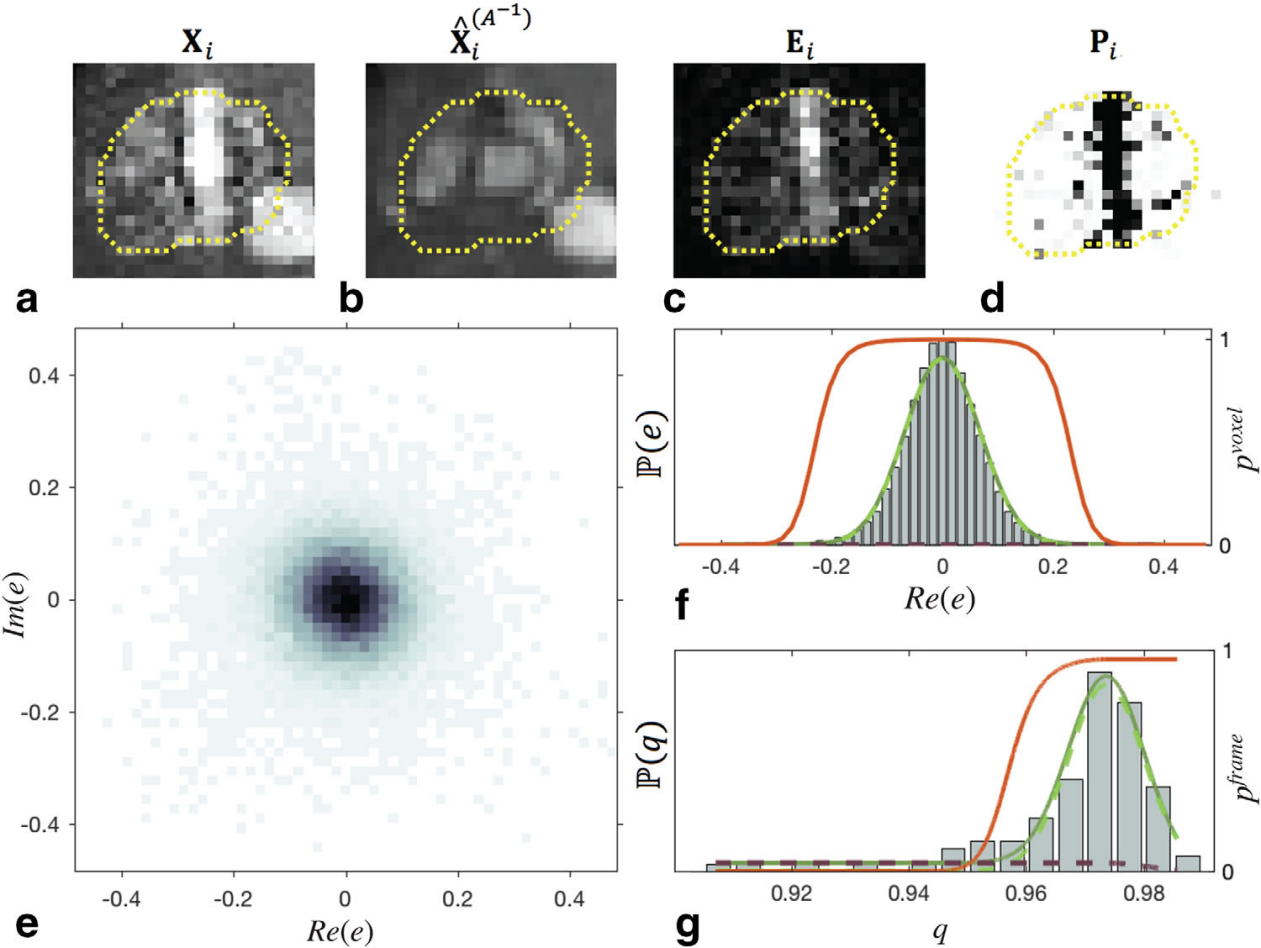
Cropped views of the heart of the fetus shown in full field of view in Figure 3a in (**a**) real‐time image frame, $X_{i}$, and (**b**) matched reference image, ${\overset{⁁}{X}}_{i}^{\left(A^{-1}\right)}$. The difference between these two images yields (**c**) an error map, $E_{i}$, from which (**d**) a probability map, $P_{i}$, is generated. Voxel‐wise probabilities are determined from (**e**) the distribution of complex error values in the region of interest (dotted line) in (c) for all image frames, for which (**f**) a cross‐section with Im(e) = 0 is shown to aid interpretation. The voxel‐wise likelihood of observing errors P(e) (solid green line) is modeled using a mixture of a Gaussian inlier distribution (dashed green line) and uniform outlier distribution (dashed purple line), to give voxel‐wise posterior probability weighting $p^{voxel}$ (red line). Frame‐wise probabilities are determined in a similar manner based on (**g**) the distribution of frame potentials, $q_{i}$, using a mixture of a Rician inlier distribution (dashed green line), $R(1-q_{i})$, and a uniform outlier distribution (dashed purple line), resulting in frame‐wise posterior probability weighting $p^{frame}$ (red line). Signal intensities of complex‐valued voxels are shown in (a), (b), and (c) for visualization. The gray scale of the probability map in (**d**) varies from 0 (black) to 1 (white).

Robust statistics were employed to exclude inconsistent data using an approach that has been shown to be effective for reconstruction of volumetric fetal MRI from 2D images 26. Each voxel and frame was classified as an inlier or outlier using mixture models of the two classes, with parameters estimated using expectation maximization. In this way, the weighting of voxels that were corrupted by motion artifact and frames that were misaligned or contained inconsistent anatomical views could be reduced or rejected completely. The elements of probability maps $P_{i}$ were calculated as the product of voxel‐ and frame‐wise probabilities, $p_{j,i}=p_{j,i}^{voxel}\;p_{i}^{frame}$. Figure 4 depicts the voxel‐ and frame‐wise outlier rejection process.

Voxel‐wise classification was based on voxel‐wise error maps $E={\left\{E_{i}\right\}}_{i=1,\ldots ,N_{i}}$, calculated as

(8) $$E_{i}=X_{i}-{\overset{⁁}{X}}_{i}^{\left(A^{-1}\right)},$$

where the inverse spatial transformation, $A^{-1}$, was used to align the voxels in each reference image frame ${\overset{⁁}{X}}_{i}$ with the acquired data in $X_{i}$ (Fig. 4 a‐c).

The likelihood of observing error $e_{j,i}$ was modeled as

(9) $$P(e_{j,i}|\sigma_{e},c_{e})=G(e_{j,i}|\sigma_{e})c_{e}+b_{e}(1-c_{e})$$

using a mixture of a bivariate Gaussian inlier distribution $G(e_{j,i}|\sigma_{e})$ with zero mean and variance $\sigma_{e}$ in both real and imaginary components (Fig. 4e), and a uniform outlier distribution of density $b_{e}$ with a mixing proportion given by $c_{e}$ (Fig. 4f). These distribution parameters were estimated by maximizing the log‐likelihood $\underset{j\in ROI}{\sum }\underset{i}{\sum }log\;P(e_{j,i}|\sigma_{e},c_{e})$, resulting in an estimate of the voxel‐wise posterior probability given by

(10) $$p_{j,i}^{voxel}=\frac{G(e_{j,i}|\sigma_{e})c_{e}}{G(e_{j,i}|\sigma_{e})c_{e}+b_{e}(1-c_{e})}$$

Frame‐wise posterior probability was used to further reduce the weighting of frames containing many voxels with low voxel‐wise posterior probability. The frame‐wise potential, $q_{i}$, of each real‐time image $X_{i}$ was calculated as $q_{i}=\sqrt{\underset{j\in ROI}{\sum }{\left(\;p_{j,i}^{voxel}\;\right)}^{2}/N_{j\in ROI}}$, where $N_{j\in ROI}$ is the number of voxels in the ROI. The likelihood of observing $q_{i}$ was modeled as the mixture of a Rician inlier distribution, $R(1-q_{i}|1-\nu_{q},\sigma_{q})$ with noncentrality $\nu_{q}$ and scale $\sigma_{q}$, and uniform outlier distribution with density $b_{q}$, given by

(11) $$P(q_{i}|\nu_{q},\sigma_{q},c_{q})=R(1-q_{i}|1-\nu_{q},\sigma_{q})c_{q}+b_{q}(1-c_{q}),$$

with mixing proportion given by $c_{q}$ (Fig. 4g). In practice, the right tail of the frame‐wise outlier class was tapered to ensure $p_{i}^{frame}$ was nondecreasing. As with voxel‐wise outlier rejection, distribution parameters were estimated by log‐likelihood maximization to give frame‐wise posterior probability

(12) $$p_{i}^{frame}=\frac{R(1-q_{i}|1-\nu_{q},\sigma_{q})c_{q}}{R(1-q_{i}|1-\nu_{q},\sigma_{q})c_{q}+b_{q}(1-c_{q})}$$

### Cardiac Cine Reconstruction

For each iteration, m, of the full pipeline (Fig. 1), a cine image series, $Y^{\left[m\right]}={\left\{Y_{\vartheta_{k}}^{\left[m\right]}\right\}}_{k=1,\ldots ,N_{k}}$, was generated for $N_{k}$ = 25 uniformly distributed cardiac phases using Equation [5]. To aid visualization of subtle features, reconstructed spatial resolution was increased in $Y_{\vartheta_{k}}$ by first increasing the spatial resolution of real‐time images $X_{i}$ and probability maps $P_{i}$. Real‐time images were zero‐padded in k‐space to an apparent resolution of 1.25 × 1.25 mm to improve visualization of small structures 27. No k‐space apodization was applied to preserve spatial resolution. Gibbs ringing effects were reduced when combining spatially transformed real‐time images with subvoxel displacements. Probability maps were scaled to the same resolution using linear interpolation to maintain discontinuities.

Processing continued iteratively until the algorithm converged or $N_{m}$ = 5 iterations were reached.

Convergence was measured as the root mean square difference (RMSD) of the signal intensity in the ROI of successive cardiac cine reconstructions, $RMSD{\left(|Y^{\left[m\right]}|,|Y^{\left[m-1\right]}|\right)}_{j\in ROI}<\delta_{\left|Y\right|}$. After some experimentation, the convergence tolerance, $\delta_{\left|Y\right|}$, was set to 0.1% of the maximum signal intensity in the real‐time images within the ROI.

### Evaluation

Line profiles in *x‐t*, drawn across the cardiac anatomy, were used to visualize the signal evolution during the cardiac cycle of the real‐time and cine images series, and to assess the effect of motion correction and outlier rejection. Voxel‐ and frame‐wise probabilities used for outlier rejection were visualized and inspected in the real‐time images for correspondence between outlier classification and visually inconsistent voxels and frames. The effect of *k‐t* SENSE regularization was assessed by comparing cine image series generated from real‐time images reconstructed using spatially uniform (Eq. [1]) and spatially adaptive (Eq. [2]) regularization.

Image quality was assessed by two fetal cardiologists (D.L., K.P.). Cine image series reconstructed using the proposed method were scored on a 5‐point scale for both gross and fine features of the fetal heart and great vessels—score 4: high contrast between blood and myocardium, distinct appearance of fine structural details, and no noteworthy artifacts; score 3: adequate image quality to determine most intracardiac structures, some insignificant artifacts; score 2: sufficient image quality to determine some intracardiac structures, despite some significant artifacts; score 1: some significant artifacts, adequate image quality to determine general ventricular morphology only; and score 0: inadequate image quality to visualize global cardiac structure. The two reviewers’ assessments were combined as an average score.

The utility of each step in the pipeline was evaluated by comparing the results of the full pipeline with those from reduced pipelines that excluded one or more steps. An entropy‐based image quality metric was used to compare the resulting cine image series. The entropy metric favors high contrast and has been shown to be sensitive to motion artifacts in MRI 28. The entropy, H, of cine image series

(13) $$H\left(Y\right)=\underset{j\in ROI}{\sum }\underset{k}{\sum }\frac{\left|y_{j,\vartheta_{k}}\right|}{y_{max}}ln\left(\frac{\left|y_{j,\vartheta_{k}}\right|}{y_{max}}\right)$$

where signal intensities $\left|y_{j,\vartheta_{k}}\right|$ were normalized by $y_{max}=\sqrt{\underset{j\in ROI}{\sum }\underset{k}{\sum }|y_{j,\vartheta_{k}}{|}^{2}}$. Relative entropy was used for comparison between data sets, where entropy values were normalized by dividing by the entropy of the cine generated from initial parameter values, $H(Y^{\left[0\right]})$.

## RESULTS

**Figure 5:**
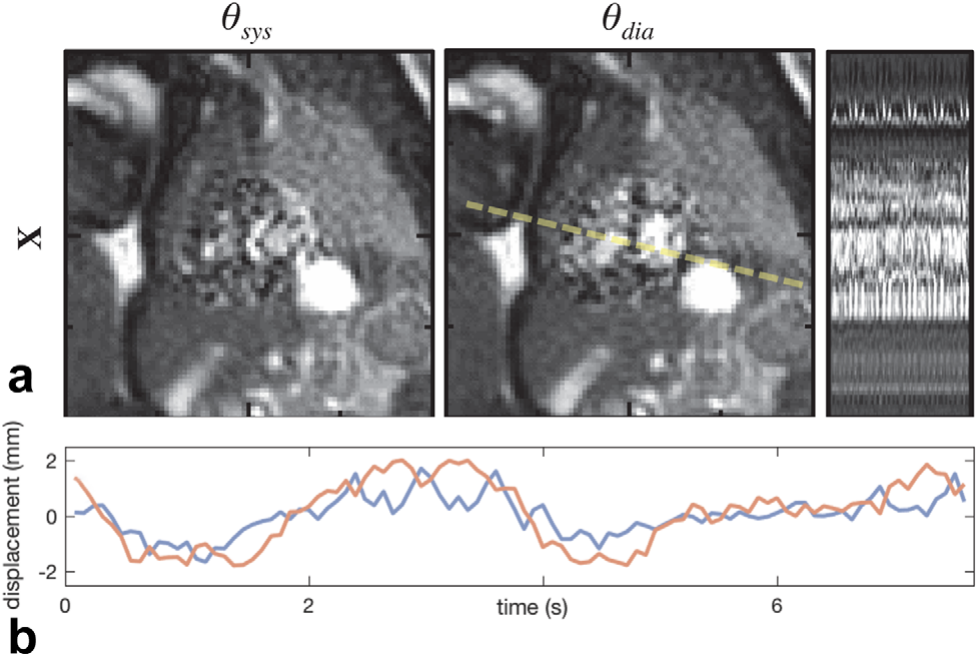
Reconstructed real‐time image series for fetus shown in full field‐of‐view image in Figure 3a. (**a**) Reconstructed real‐time images, X, showing cropped views of the fetal heart at end‐ventricular systole, $\theta_{sys}$, and diastole, $\theta_{dia}$, with line profile across ventricles (dashed line) showing real‐time frames ordered based on estimated cardiac phase. (**b**) Mean in‐plane displacement of voxels j∈region of interest of $X^{\left(A\right)}$ in vertical (red line) and horizontal (blue line) directions showing a pattern of displacement consistent with the effects of maternal respiration. Cropped views cover 100 mm in each direction, with 25‐mm markers shown for reference.

**Figure 6:**
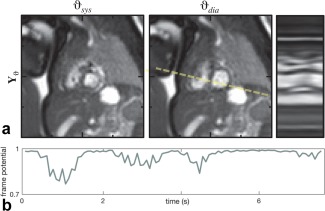
Cine image series, Y, reconstructed from real‐time image series shown in Figure 5a. (**a**) Cropped views of the fetal heart in cine image frames at systole, $\vartheta_{sys}$, and diastole, $\vartheta_{dia}$, with line profile across the ventricles (dashed line) showing the temporal dynamics of the cardiac anatomy. (**b**) Frame potential, $q_{i}$, of real‐time image frame $X_{i}$ was reduced during periods of large displacement, shown in Figure 5b, as the fetal heart was also displaced through‐plane. Cropped views cover 100 mm in each direction, with 25‐ mm markers shown for reference.

Data was acquired in 30 singleton pregnancies, including nine cases with two scans in different orientations in the same fetus. Of these 39 data sets, three were acquired during periods of general fetal movement, when the fetus was in motion during most of the scan, precluding further processing. The remaining 36 data sets were successfully reconstructed using the method depicted in Figure 1. An example reconstruction is shown in Figures 5 and 6.

Spatiotemporal sparsity similar to the pattern shown in Figure 2 was evident in all data sets. An example real‐time image frame, acquired without maternal breath‐hold, is shown in Figure 3a, in which a high level of noise can be observed within the ROI. In all cases, the estimated heart rate resulted in a cardiac phase reordering that revealed the pulsatility of the fetal heart in a combined cardiac cycle, similar to the *x‐t* profile shown to the right of Figure 5a. In three of the cases with the two data sets in the same fetus, the scans were performed in quick succession such that little change in heart rate was anticipated between them. In these cases, the estimated heart rates differed by 1.4, 1.5, and 2.0% of the mean estimated heart rate, respectively, providing support for the use of a single heart rate for cardiac synchronization of data from each short acquisition. In most of the data sets, the estimated heart rate did not change between iterations of the pipeline. However, the alignment of the fetal heart across real‐time images resulting from motion correction had the effect of making the peaks in the *x‐f* signal more prominent, and in two data sets a difference of more than 1% of the final heart rate was observed between initial and final iterations.

Figure 5b shows mean in‐plane displacement in $X_{i}^{\left(A\right)}$ for voxels j∈ROI plotted against time with a pattern of displacement consistent with the effects of maternal respiration. The frame potential, shown in Figure 6b, was reduced during periods of large displacement because the fetal heart was also displaced through‐plane. The maximum in‐plane displacement in this data set was 2.1 mm, and 27% of voxels were considered outliers ( $p_{j\in ROI,i}<0.5$). The maximum in‐plane displacement in all data sets ranged from 0.5 to 2.3 mm, with a median of 1.8 mm, whereas a median of 13% of voxels were considered outliers, ranging from 2% to 63%. Frames classified as outliers were typically those misaligned or heavily corrupted due to motion. The final reconstructed cine series (Fig. 6a) shows clear depiction of the cardiac anatomy.

**Figure 7:**
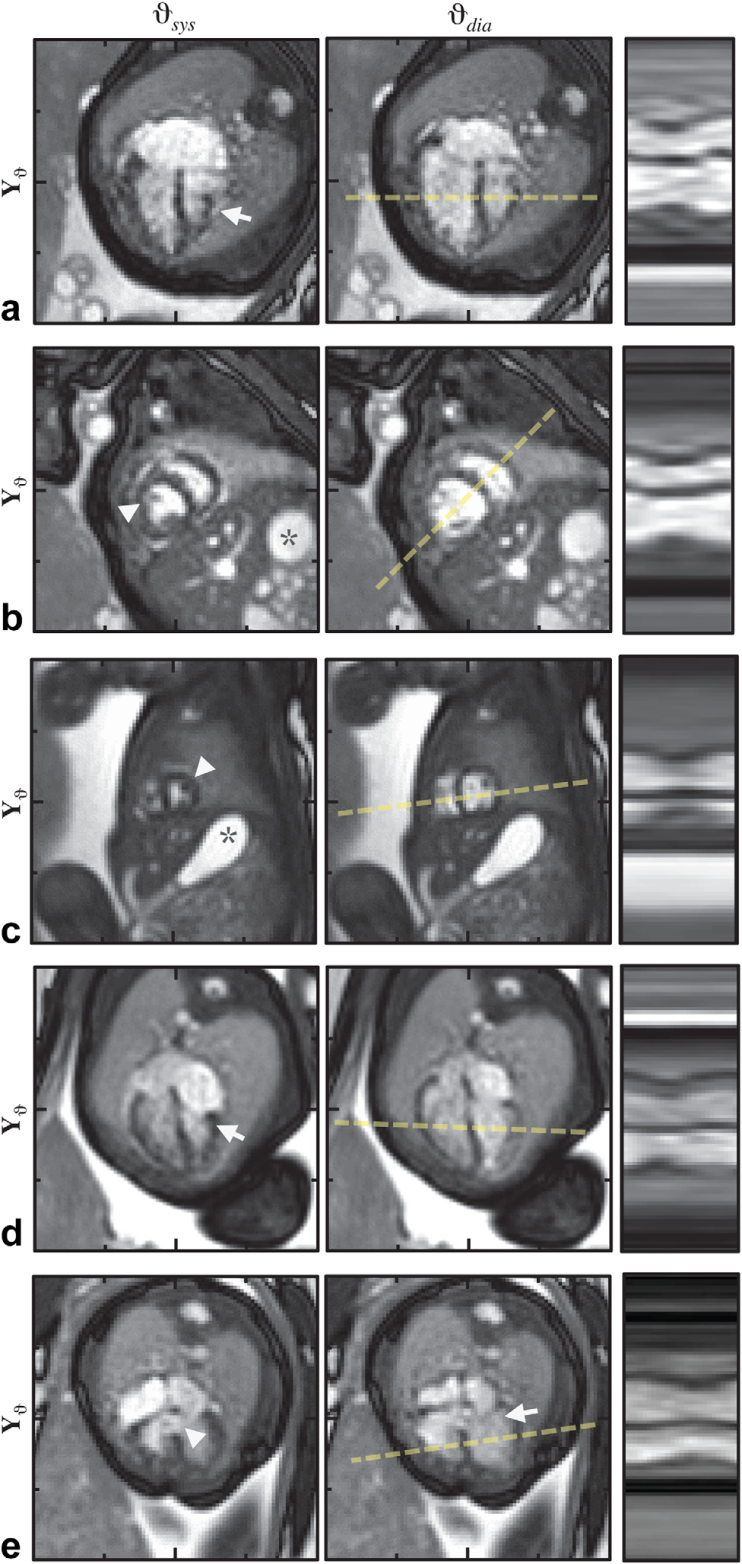
Reconstructed cine image series, Y, with heart shown at systolic, $\vartheta_{sys}$, and diastolic, $\vartheta_{dia}$, cardiac phases and line profile time plots corresponding to dashed line across the ventricles. Cropped views cover 100 mm in each direction, with 25‐mm markers shown for reference. The heart of a 33‐week gestational‐age fetus with atrioventricular and ventriculoarterial discordance is shown in (**a**) long‐ and (**b**) short‐axis orientations revealing transposed morphological left and right ventricles. The moderator band (arrow) can be seen on the morphological right ventricle in the anatomical left/anterior position in four‐chamber view of the heart with apex to the left in (a), whereas mitral‐valve papillary muscles (arrowhead) can be seen on the morphological left ventricle on the anatomical right side opposite the stomach (asterisk) in short axis view in (b). (**c**) Short axis view in 27‐week gestational age fetus with normal situs, for comparison with (b), in which papillary muscles (arrowhead) are seen on the morphological left ventricle on the anatomical left side with the stomach (asterisk) for reference. (**d**) Four‐chamber view in a 33‐week gestational age fetus with coarctation of the aorta. (**e**) Long‐axis view in a 30‐week gestational age fetus with a ventricular septal defect (arrowhead). Atrioventricular valves (arrows) can be seen in (d) and (e).

Expert image evaluation resulted in a median combined score of 2.5 out of 4. No individual cine image series was given a score of 4, although 89% of combined scores were higher than 1, indicating that intracardiac structures as well as general ventricular morphology could be determined in most of the cases. Abnormalities suspected from echocardiographic examination prior to MRI were confirmed when appropriate imaging planes were acquired, as shown in the cine reconstructions in Figure 7.

**Figure 8:**
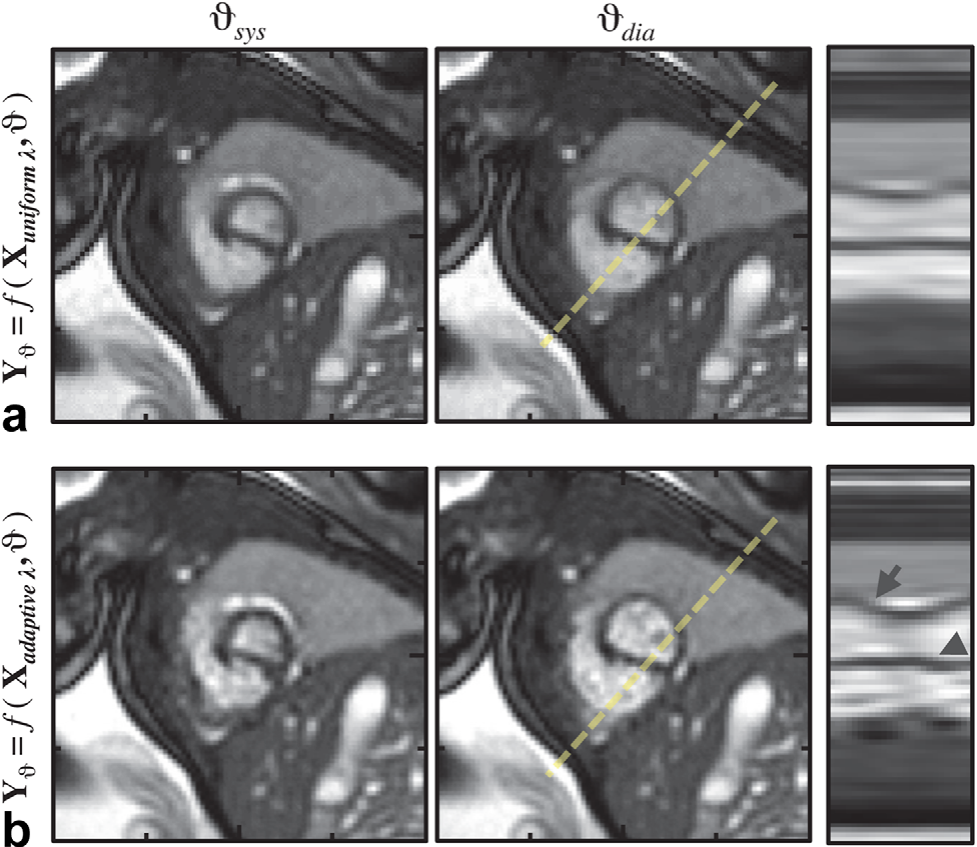
Comparison of (**a**) cine images, $Y_{\vartheta }$, reconstructed from real‐time image series, $X_{uniform\lambda }$, generated using spatially uniform regularization (Eq. [1]) with $\lambda =\lambda_{0}$, and (**b**) cine images reconstructed from real‐time image series, $X_{adaptive\lambda }$, generated using spatially adaptive regularization (Eq. [2]), in 33‐week gestational age fetus. The impact of *k‐t* SENSE regularization on reconstructed cine images can be seen in the additional high‐frequency temporal dynamics in the line profiles (dashed lines), including pulsatility (arrow) and end‐systolic torsion (arrowhead). Cropped views cover 100 mm in each direction, with 25‐mm markers shown for reference.

**Figure 9:**
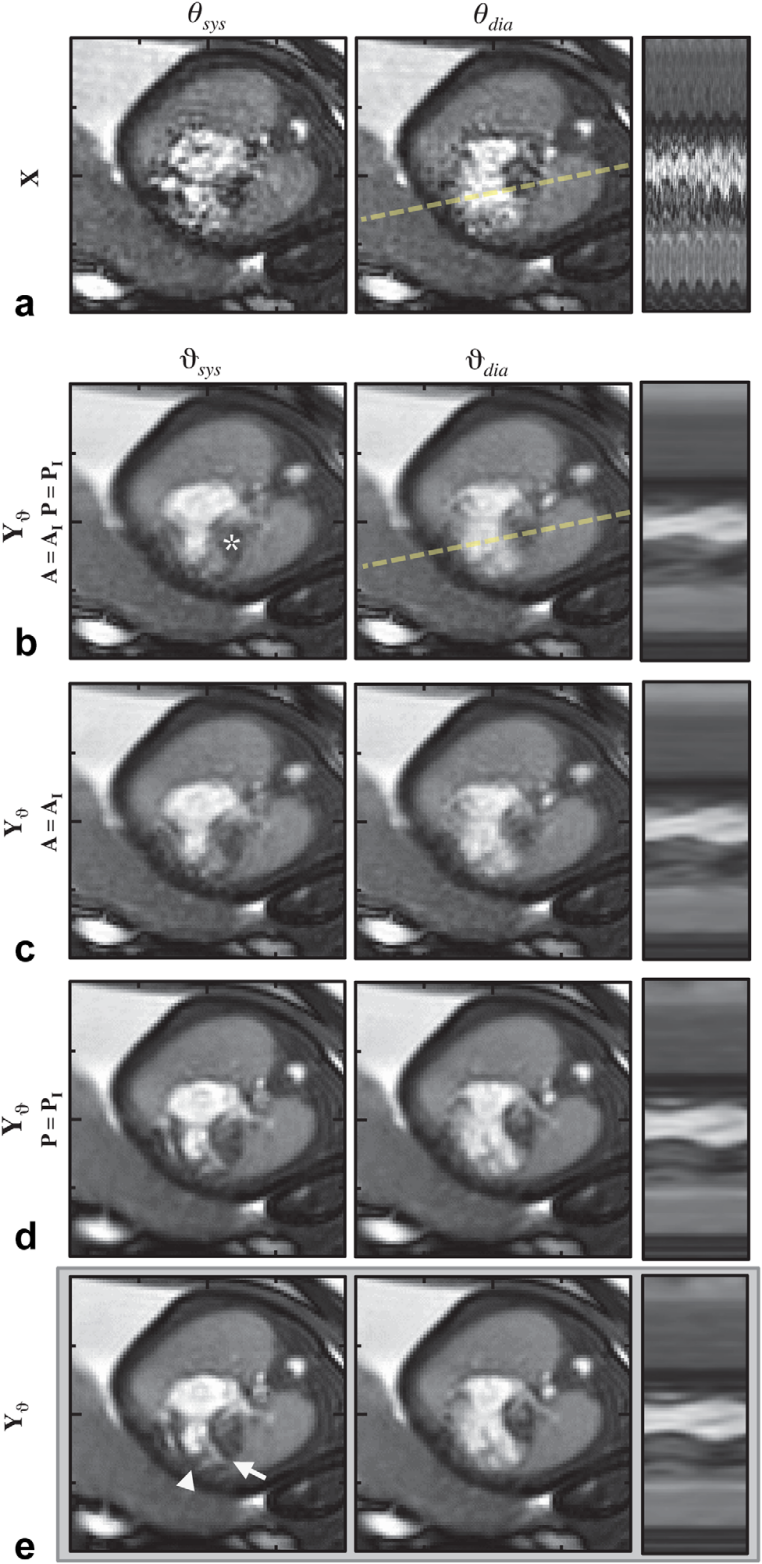
Comparison of cine image series reconstructed using some or all steps in the proposed pipeline. Cropped views of the heart of a 30‐week gestational age fetus with hypoplastic left‐heart syndrome at systole and diastole reveal contraction and dilation of the right ventricle in a line‐profile time plot corresponding to the dashed line across the ventricles for (**a**) reconstructed real‐time images X reordered based on cardiac phase. Cine image series, Y, reconstructed using (**b**) cardiac synchronization only, with $A=A_{I}$ and $P=P_{I}$, (**c**) cardiac synchronization and outlier rejection, with $A=A_{I}$, (**d**) cardiac synchronization and motion correction, with $P=P_{I}$, and (**e**) the full pipeline show the heart, including underdeveloped left ventricle (asterisk), with added detail. Each step included in the pipeline increased the clarity of blood–tissue boundaries in the resulting reconstructed cine images, improving depiction of small anatomical features such as the interventricular septum (arrow) and moderator band (arrowhead). Cropped views cover 100 mm in each direction, with 25‐mm markers shown for reference.

**Figure 10:**
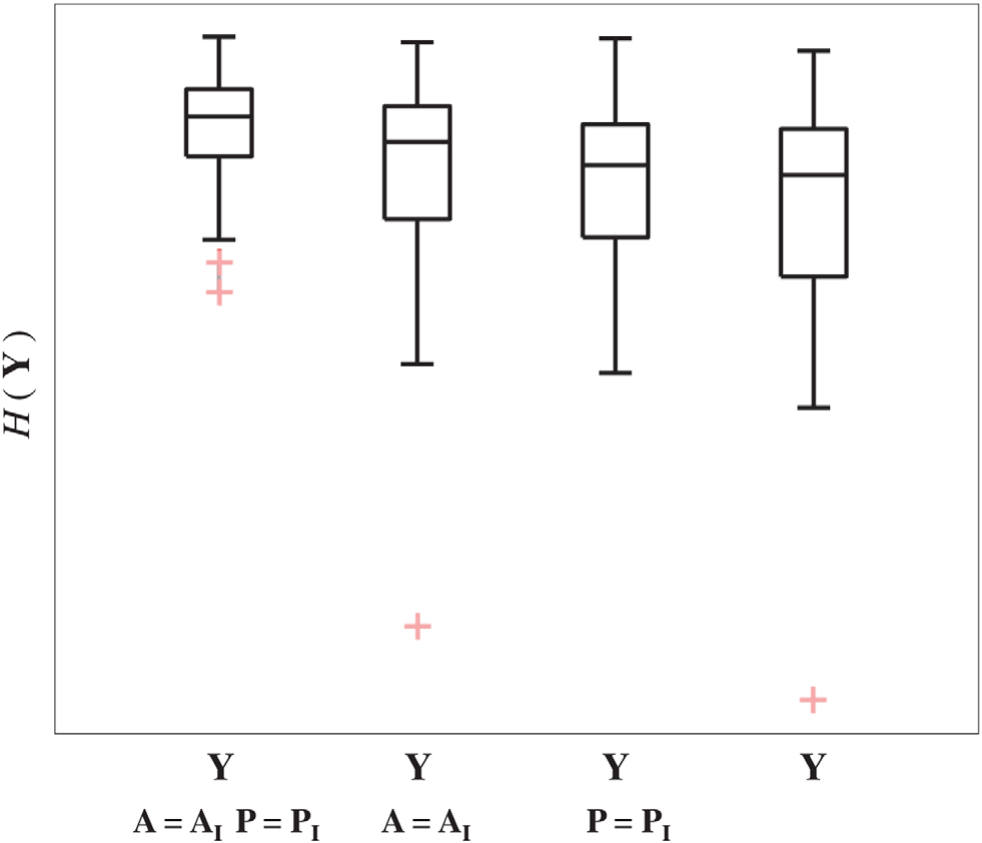
Comparison of image quality in cine image series, Y, reconstructed using cardiac synchronization only ( $A=A_{I}$, $P=P_{I}$), cardiac synchronization and outlier rejection ( $A=A_{I}$), cardiac synchronization and motion correction ( $P=P_{I}$), and the full pipeline. The greatest improvement in image quality, that is, reduction in relative entropy H(Y), in all 36 data sets was achieved using the combination of steps in the proposed pipeline. Boxes span the first to third quartiles with a band at the median. Whiskers extend to the furthest value within 1.5 interquartile range.

The utility of each step in the full method was evaluated by comparing the cine image series reconstructed using the full pipeline with those reconstructed leaving out one or more steps. Figure 8 illustrates how the adaptive regularization in the *k‐t* SENSE reconstruction allows additional details of the cardiac motion to be depicted. An example is shown in Figure 9, in which the cine image series reconstructed using the full pipeline had improved anatomical depiction compared to those reconstructed using a subset of the pipeline. Figure 10 presents the quantitative results for all cases, showing that each step in the reconstruction served to improve image quality. Of the two main correction steps, the largest reduction in relative entropy was due to motion correction in 67% of the reconstructions and due to outlier rejection in the other 33%.

Videos of the real‐time and cine image series shown in Figures 7 to 9 are available as Supporting Videos S1 to S3.

## DISCUSSION

Highly accelerated real‐time imaging was able to capture the motion of the fetal heart as well as the surrounding anatomy, allowing for direct estimation of, and correction for, cardiac pulsation and motion—as well as rejection of data that could not be made consistent both at the individual voxel and frame level. Cine reconstruction effectively combined the data as a single cardiac cycle, increasing the visibility of small, dynamic anatomical features, and enabled visualization of congenital abnormalities in the clinical cases. The use of robust statistics enabled the reconstruction to effectively deal with inconsistencies in the data that could not be resolved by in‐plane motion correction, such as due to maternal respiration, motion artifact, and short episodes of general fetal movement. The full pipeline was found to be highly effective, with successful reconstructions in all but three data sets in which there was fetal motion, such that there were few frames depicting a single consistent cardiac cross‐sectional slice.

The reordered real‐time images produced cine sequences that were sampled far more densely in cardiac phase than the temporal resolution of the individual image frames. This allowed images to be compounded without sacrificing temporal resolution by using a tuned weighting function that respected sequence timing and cardiac period. Because image‐compounding recovered SNR, it was feasible to preserve the full temporal resolution of the undersampled acquisition by selectively regularizing the *k‐t* SENSE reconstruction to favor temporal fidelity in the fetal heart. This was achieved using Equation [2], exploiting the disparity in dynamic content between the fetal heart and the rest of the imaged scene. Despite the low SNR of the resulting time series of real‐time images, heart rate estimation, motion correction, and outlier rejection steps all were found to perform reliably, with no failures. The resulting postprocessing pipeline is fully automatic, except for a single manual step in which a region of interest covering the fetal heart is defined. It may be possible to identify an appropriate ROI using an image segmentation approach, resulting in a fully automatic reconstruction pipeline, although this was outside the scope of the present study.

The spatial and temporal resolution achieved in this work improves on the resolution in previous studies of the fetal heart using dynamic MRI 24, 29. The proposed method preserves temporal fidelity at the acquired resolution because there effectively is no regularization in the region of the fetal heart in the spatially adaptive *k‐t* SENSE reconstruction. Cardiac anatomy and motion is clearly depicted in the resulting cine images such that it may be possible to characterize the myocardium and relative chamber sizes at the current resolution; however, a higher resolution would be desirable for accurate functional assessment based on guidelines for ventriculography in infants: 1.2 × 1.2 × 4.0 mm, $t_{RR}/15$ 30. Fetal cardiac imaging using compressed sensing reconstruction of undersampled segmented cine data to achieve high resolution and short scan times has recently been reported 13, although the regularized reconstruction can impact spatial and temporal resolution at high acceleration rates. Thus, a direct comparison with the results reported here is not simple. Radial sampling is compatible with the proposed framework but is less well matched to image‐domain processing with locally reduced regularization at the heart, as used in this study, because it results in a less sparse *x‐f* alias distribution and is less efficient than Cartesian coverage due to dense sampling around the k‐space origin. Functional assessment using the proposed method remains to be explored, likely in combination with further development and optimization of the acquisition and reconstruction strategy to improve resolution and include multislice data to allow for volumetric reconstruction.

It is a challenge to prospectively account for each source of motion effecting the position of the fetal heart. Although segmented cine acquisition potentially can achieve both higher spatial and cardiac phase resolution, the use of a dynamic acquisition offers potential advantages for addressing both motion and artifacts. The ability to process data in image‐space allowed for spatial and temporal operations to be applied to the fetal heart in isolation so that incidental changes in other parts of the field of view, such as due to movement of the mother or peripheral parts of the fetal anatomy, did not influence the result.

The focus of this work was to demonstrate that dynamic fetal cardiac MRI can form the basis of an effective imaging approach in the presence of maternal and fetal motion, and to develop the required processing pipeline. The starting point was the concept of adaptive reconstruction of undersampled dynamic imaging, required to achieve a temporal resolution fast enough to freeze the fetal heart. In this study, low‐resolution training data was acquired to guide the reconstruction, effectively doubling the acquisition time, which is both inefficient and increases the possibility of data corruption due to general fetal movement. In the future, several improvements could be investigated. Integration of autocalibrated (ie, reference‐less) dynamic image reconstruction 31, 32 with the proposed method could improve motion robustness because it reduces scan duration and ensures that the priors used in reconstruction are consistent with the target data, but it is likely to reduce temporal resolution. A calibration‐free 33 reconstruction, leveraging knowledge of the spatial location of dynamic regions and expected bandwidths, also may be possible. Additional temporal filtering effects may be reduced by appropriate filtering of the subtracted baseline signal 34, which will also be of benefit if coil sensitivities are autocalibrated instead of derived from a prescan, as they are currently. In this work, one set of adaptive regularization parameters was used to reconstruct real‐time images using Equation [2] for all data sets because these parameters are independent of signal level. A better balance of tradeoffs between noise suppression and temporal fidelity potentially could be achieved by tuning these parameters case by case, such as by an automated L‐curve analysis.

The use of a constant heart rate for cardiac synchronization leads to timing errors because the true heart rate varies from beat to beat. In this study, a small number of cases were used to check that mean fetal heart rate is stable over the duration of the acquisition by acquiring multiple data sets in quick succession. However, timing errors within a data set would still be expected. To explore the potential magnitude of this effect, Monte Carlo simulations of fetal heart rate traces were generated using a previously established model 35, with expected distribution of baseline fetal heart rate 5 and beat‐to‐beat variation 6. Based on 500 runs, each simulating approximately 8 seconds with measurements every 71 ms to 88 ms (96 measurements total), cardiac phase errors, $\Delta \theta_{i}$, are expected to be within $\pm \;0.10\;t_{RR}$ 95% of the time and within $\pm \;0.05\;t_{RR}$ 75% of the time. These errors are small in comparison to the image acquisition time. A refinement of the method could be to incorporate an appropriate self‐gating technique 36, 37 to accommodate beat‐to‐beat variation, thus reducing timing error. This might become important if longer acquisition durations are used.

Motion correction and outlier rejection steps were shown to improve the image quality of the final reconstructed cine images in all cases. In two‐thirds of the datasets, reconstructed motion correction alone provided more improvement in image quality than outlier rejection alone. However, the two processes proved to be complementary, with the combination of the two providing more improvement in image quality than either process alone.

The proposed method shows promise as a framework for comprehensive fetal cardiac MRI. Working in the image domain allowed separation of fetal cardiac pulsation from other extraneous changes elsewhere in the field of view, which get mixed together when processing is done in k‐space. In the future, this work may be extended to multislice acquisition to reconstruct volumetric data, offering the potential for fully motion‐compensated 3D assessment of dynamic features of the fetal heart and great vessels.

## Supporting information

Additional supporting information may be found in the online version of this article.

**Video S1**. Videos of dynamic and cine image series shown in Figure 7. Real‐time images are shown in full field of view (left) and cropped view (center) at acquired frame rate, with cropped view of cine image series (right) looped for duration of video. The full field of view is 400 × 304 mm and cropped views are 100 × 100 mm. Images are oriented with the fetus in radiographic image orientation.

**Video S1b**.

**Video S1c**.

**Video S1d**.

**Video S1e**.
(**a**) Thirty‐three week gestational age fetus with atrioventricular and ventriculoarterial discordance shown in Figure 7a. (**b**) Thirty‐three week gestational age fetus with atrioventricular and ventriculoarterial discordance shown in Figure 7b. (**c**) Twenty‐seven week gestational age fetus shown in Figure 7c. (**d**) Thirty‐three week gestational age fetus shown in Figure 7d.(e) Thirty week gestational age fetus with ventricular septal defect shown in Figure 7e.

**Video S2**. Videos of dynamic and cine image series shown in Figure 8 showing impact of *k‐t* SENSE regularization in 33 week gestational age fetus. Real‐time image series (left) reconstructed with spatially‐uniform (top) and spatially‐adaptive regularization (bottom) are shown at acquired frame rate. Additional high‐frequency temporal dynamics can be seen between resulting cine image series (right). Cropped views are 100 × 100 mm, and images are oriented with the fetus in radiographic image orientation.

**Video S3**. Videos of dynamic and cine image series shown in Figure 9 comparing cine images reconstructed using some or all steps in the proposed pipeline in a 30 week gestational age fetus with hypoplastic left heart syndrome. Real‐time image series (left) was used to generate cines using (left to right) cardiac synchronization only; cardiac synchronization and outlier rejection; cardiac synchronization and motion correction; and the full pipeline. Cropped views are 100 × 100 mm, and images are oriented with the fetus in radiographic image orientation.

## References

[1] Votino C , Jani J , Damry N , Dessy H , Kang X , Cos T , Divano L , Foulon W , De Mey J , Cannie M . Magnetic resonance imaging in the normal fetal heart and in congenital heart disease. Ultrasound Obstet Gynecol 2012;39:322–329. 21837757 10.1002/uog.10061

[2] Loomba RS , Chandrasekar S , Shah PH , Sanan P . The developing role of fetal magnetic resonance imaging in the diagnosis of congenital cardiac anomalies: a systematic review. Ann Pediatr Cardiol 2011;4:172–176. 21976881 10.4103/0974-2069.84665 PMC3180979

[3] Dong S‐Z , Zhu M , Li F . Preliminary experience with cardiovascular magnetic resonance in evaluation of fetal cardiovascular anomalies. J Cardiovasc Magn Reson 2013;15:40. 23692653 10.1186/1532-429X-15-40 PMC3666966

[4] Schneider C , McCrindle BW , Carvalho JS , Hornberger LK , McCarthy KP , Daubeney PEF . Development of Z‐scores for fetal cardiac dimensions from echocardiography. Ultrasound Obstet Gynecol 2005;26:599–605. 16254878 10.1002/uog.2597

[5] Pildner von Steinburg S , Boulesteix A‐L , Lederer C , Grunow S , Schiermeier S , Hatzmann W , Schneider K‐TM , Daumer M . What is the “normal” fetal heart rate? PeerJ 2013;1:e82. 23761161 10.7717/peerj.82 PMC3678114

[6] Ortiz MR , Aguilar SD , Alvarez‐Ramirez J , Martínez A , Vargas‐Garcia C , González‐Camarena R , Echeverría JC . Prenatal RR fluctuations dynamics: detecting fetal short‐range fractal correlations. Prenat Diagn 2006;26:1241–1247. 17139696 10.1002/pd.1595

[7] Vadeyar SH , Moore RJ , Strachan BK , Gowland PA , Shakespeare SA , James DK , Johnson IR , Baker PN . Effect of fetal magnetic resonance imaging on fetal heart rate patterns. Am J Obstet Gynecol 2000;182:666–669. 10739527 10.1067/mob.2000.103938

[8] Patrick J , Challis J . Measurement of human fetal breathing movements in healthy pregnancies using a real‐time scanner. Semin Perinatol 1980;4:275–286. 7001636

[9] Hayat TTA , Nihat A , Martinez‐Biarge M , McGuinness A , Allsop JM , Hajnal JV , Rutherford MA . Optimization and initial experience of a multisection balanced steady‐state free precession cine sequence for the assessment of fetal behavior in utero. Am J Neuroradiol 2011;32:331–338. 21087938 10.3174/ajnr.A2295 PMC7965695

[10] Piontelli A . Development of Normal Fetal Movements. Milan, Italy: Springer Milan; 2015.

[11] Paley MNJ , Morris JE , Jarvis D , Griffiths PD . Fetal electrocardiogram (fECG) gated MRI. Sensors (Basel) 2013;13:11271–11279. 23979479 10.3390/s130911271 PMC3821351

[12] Roy CW , Seed M , van Amerom JF , Al Nafisi B , Grosse‐Wortmann L , Yoo SJ , Macgowan CK . Dynamic imaging of the fetal heart using metric optimized gating. Magn Reson Med 2013;70:1598–1607. 23382068 10.1002/mrm.24614

[13] Roy CW , Seed M , Macgowan CK . Accelerated MRI of the fetal heart using compressed sensing and metric optimized gating. Magn Reson Med 2016:1–11. 10.1002/mrm.26290 27254315

[14] Yamamura J , Kopp I , Frisch M , Fischer R , Valett K , Hecher K , Adam G , Wedegärtner U . Cardiac MRI of the fetal heart using a novel triggering method: initial results in an animal model. J Magn Reson Imaging 2012;35:1071–1076. 22246623 10.1002/jmri.23541

[15] Yamamura J , Schönnagel B , Tavares De Sousa M , Much C , Ueberle F , Adam G , Kording F . Fetal cardiac MRI and left ventricular function assessment using a new gating strategy based on Doppler Ultrasound: Preliminary results. In Proceedings of the 23rd Annual Meeting of ISMRM, Toronto, Canada, 2015. p. 0632.

[16] Kellman P , Chefd’hotel C , Lorenz CH , Mancini C , Arai AE , McVeigh ER . Fully automatic, retrospective enhancement of real‐time acquired cardiac cine MR images using image‐based navigators and respiratory motion‐corrected averaging. Magn Reson Med 2008;59:771–778. 18302227 10.1002/mrm.21509

[17] van Amerom JFP , Kuklisova Murgasova M , Price AN , et al. Fetal cardiac cine imaging from super‐resolution reconstruction of highly‐accelerated real‐time MRI. In Proceedings of the 24th Annual Meeting of ISMRM, Singapore, 2016. p. 458.

[18] Tsao J , Boesiger P , Pruessmann KP . k‐t BLAST and k‐t SENSE: dynamic MRI with high frame rate exploiting spatiotemporal correlations. Magn Reson Med 2003;50:1031–1042. 14587014 10.1002/mrm.10611

[19] Patenaude Y , Pugash D , Lim K , et al. The use of magnetic resonance imaging in the obstetric patient. J Obstet Gynaecol Can 2014;36:349–355. 24798674 10.1016/s1701-2163(15)30612-5

[20] Hand JW , Li Y , Hajnal JV . Numerical study of RF exposure and the resulting temperature rise in the foetus during a magnetic resonance procedure. Phys Med Biol 2010;55:913–930. 20090188 10.1088/0031-9155/55/4/001

[21] De Wilde JP , Rivers AW , Price DL . A review of the current use of magnetic resonance imaging in pregnancy and safety implications for the fetus. Prog Biophys Mol Biol 2005;87:335–353. 15556670 10.1016/j.pbiomolbio.2004.08.010

[22] Saleem SN . Feasibility of MRI of the fetal heart with balanced steady‐state free precession sequence along fetal body and cardiac planes. AJR Am J Roentgenol 2008;191:1208–1215. 18806167 10.2214/AJR.07.3839

[23] Tsao J , Kozerke S , Boesiger P , Pruessmann KP . Optimizing spatiotemporal sampling for k‐t BLAST and k‐t SENSE: application to high‐resolution real‐time cardiac steady‐state free precession. Magn Reson Med 2005;53:1372–1382. 15906282 10.1002/mrm.20483

[24] Brugger PC . MRI of the fetal heart In: Prayer D, ed. Fetal MRI. Medical Radiology. Berlin, Germany: Springer; 2011; 247–258.

[25] Lloyd DFA , Amerom JFP van , Pushparajah K , et al. An exploration of the potential utility of fetal cardiovascular MRI as an adjunct to fetal echocardiography. Prenat Diagn 2016;36:1–10. 10.1002/pd.4912 PMC5082528 27521762

[26] Kuklisova‐Murgasova M , Quaghebeur G , Rutherford MA , Hajnal JV , Schnabel JA . Reconstruction of fetal brain MRI with intensity matching and complete outlier removal. Med Image Anal 2012;16:1550–1564. 22939612 10.1016/j.media.2012.07.004 PMC4067058

[27] Bernstein MA , Fain SB , Riederer SJ . Effect of windowing and zero‐filled reconstruction of MRI data on spatial resolution and acquisition strategy. J Magn Reson Imaging 2001;14:270–280. 11536404 10.1002/jmri.1183

[28] Atkinson D , Hill DL , Stoyle PN , Summers PE , Keevil SF . Automatic correction of motion artifacts in magnetic resonance images using an entropy focus criterion. IEEE Trans Med Imaging 1997;16:903–910. 9533590 10.1109/42.650886

[29] Fogel MA , Wilson RD , Flake A , Johnson M , Cohen D , McNeal G , Tian Z‐YY , Rychik J . Preliminary investigations into a new method of functional assessment of the fetal heart using a novel application of ’real‐time’ cardiac magnetic resonance imaging. Fetal Diagn Ther 2005;20:475–480. 16113578 10.1159/000086837

[30] Fratz S , Chung T , Greil GF , Samyn MM , Taylor AM , Valsangiacomo Buechel ER , Yoo S‐J , Powell AJ . Guidelines and protocols for cardiovascular magnetic resonance in children and adults with congenital heart disease: SCMR expert consensus group on congenital heart disease. J Cardiovasc Magn Reson 2013;15:51. 23763839 10.1186/1532-429X-15-51 PMC3686659

[31] Malik SJ , Schmitz S , O’Regan D , Larkman DJ , Hajnal JV . x‐f Choice: reconstruction of undersampled dynamic MRI by data‐driven alias rejection applied to contrast‐enhanced angiography. Magnetic Resonance in Medicine 2006;56:811–823. 16897770 10.1002/mrm.21008

[32] Ponce IP , Blaimer M , Breuer FA , Griswold MA , Jakob PM , Kellman P . Auto‐calibration approach for k‐t SENSE. Magn Reson Med 2014;71:1123–1129. 23554094 10.1002/mrm.24738

[33] Madore B , Glover GH , Pelc NJ . Unaliasing by Fourier‐encoding the overlaps using the temporal dimension (unfold), applied to cardiac imaging and fMRI. Magn Reson Med 1999;42:813–828. 10542340 10.1002/(sici)1522-2594(199911)42:5<813::aid-mrm1>3.0.co;2-s

[34] Blaimer M , Ponce IP , Breuer FA , Jakob PM , Griswold MA , Kellman P . Temporal filtering effects in dynamic parallel MRI. Magn Reson Med 2011;66:192–198. 21695723 10.1002/mrm.22795

[35] Jansz MS , Seed M , van Amerom JFP , Wong D , Grosse‐Wortmann L , Yoo S‐J , Macgowan CK . Metric optimized gating for fetal cardiac MRI. Magn Reson Med 2010;64:1304–1314. 20632406 10.1002/mrm.22542

[36] Larson AC , White RD , Laub G , McVeigh ER , Li D , Simonetti OP . Self‐gated cardiac cine MRI. Magn Reson Med 2004;51:93–102. 14705049 10.1002/mrm.10664 PMC2396326

[37] Nijm GM , Sahakian AV , Swiryn S , Carr JC , Sheehan JJ , Larson AC . Comparison of self‐gated cine MRI retrospective cardiac synchronization algorithms. J Magn Reson Imaging 2008;28:767–772. 18777546 10.1002/jmri.21514 PMC2597286

